# Medical Benefits and Polymer Applications of Grapes

**DOI:** 10.3390/polym17060750

**Published:** 2025-03-12

**Authors:** Hsiuying Wang

**Affiliations:** Institute of Statistics, National Yang Ming Chiao Tung University, Hsinchu 300093, Taiwan; wang@stat.nycu.edu.tw

**Keywords:** drug delivery, grape, medical effect, polymer, tissue engineering, wound dressing

## Abstract

Grapes are a fruit with origins dating back to ancient times. Their first recorded use, as mentioned in the Bible, was in winemaking. The abundance of bioactive compounds in grapes makes them highly valuable. So far, many varieties of cultivated grapes have been developed for table grapes, wine grapes, and raisin production. In addition to these uses, since grapes contain a variety of nutrients, including resveratrol, flavonoids (such as flavonols, anthocyanins, and catechins), melatonin, vitamins, acids, tannins, and other antioxidants, grape extracts have been widely studied for medical applications. This paper reviews the medical effects of these compounds on cancer, cardiovascular disease, brain and neurological disorders, eye diseases, skin disorders, kidney health, diabetes, and gastric diseases, along with the medical applications of grapes in drug delivery, wound dressing, and tissue engineering. In addition, the limitations of the grapes-derived polymers and future research perspectives are discussed. These benefits highlight that the value of grapes extends far beyond their traditional use in wine and raisin production.

## 1. Introduction

**Figure 1:**
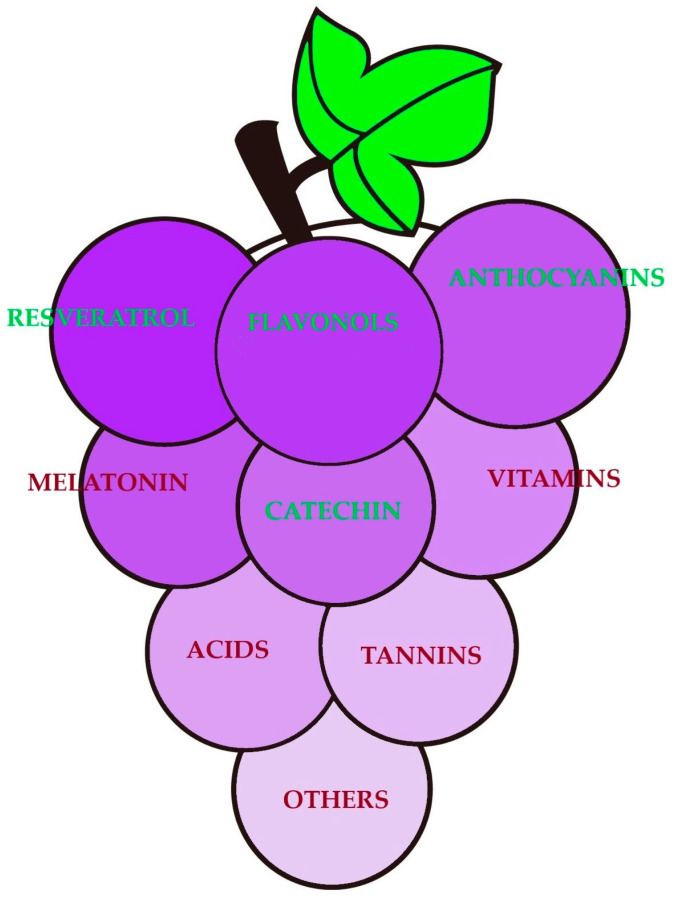
Nutritional compounds of grapes.

**Figure 2:**
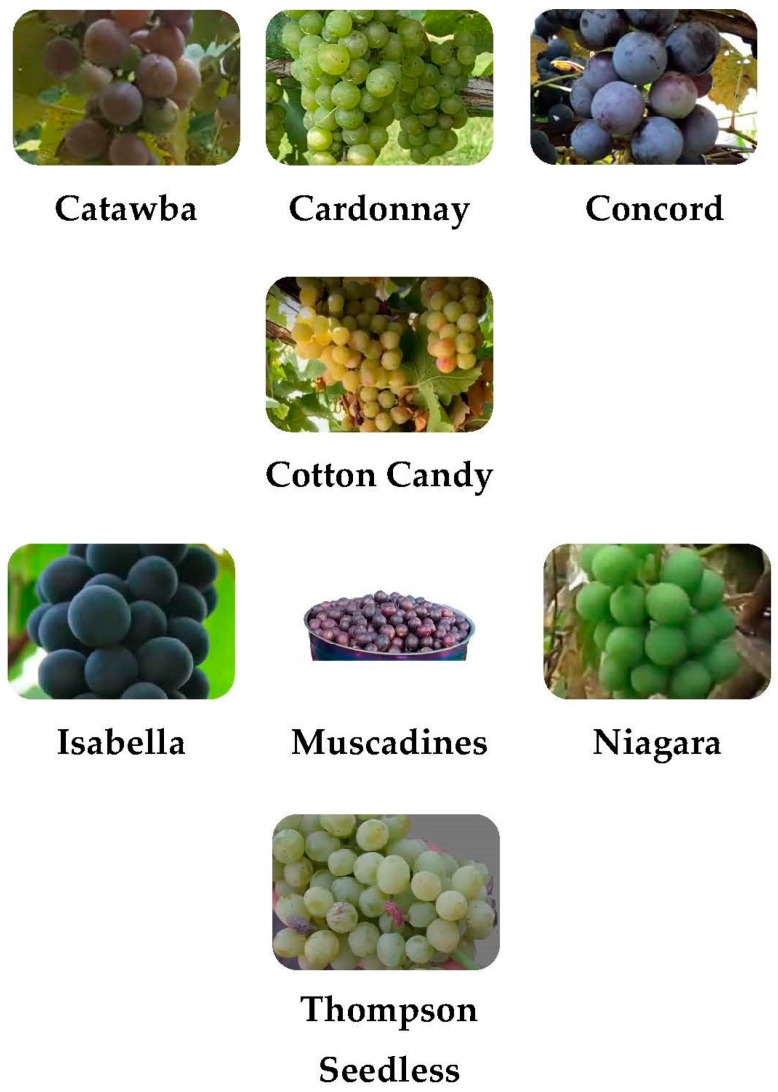
Some varieties of grapes.

Grapes are an ancient fruit with a long history, similar to olives, pomegranates, and figs [1,2]. They are mentioned in the Old Testament “And Noe, a husbandman, began to till the ground, and planted a vineyard” [3]. One of the most well-known uses of grapes is winemaking, which holds significant historical and cultural importance. Grapes are also widely consumed as raisins, grape juice, or used to make other products. Vitis vinifera grapes, along with non-vinifera varieties and hybrids, are consumed as fresh table grapes, dried into raisins, or processed into grape juice concentrate [4].

Grapes are also fermented and distilled for industrial applications and the production of wine, an alcoholic beverage [4]. Wine is considered one of the most important beverages, offering the benefits of cheering people and promoting good health, but it also has the disadvantage of causing drunkenness and acting like a poison when consumed in excessive amounts.

There are many nutritional compounds in grapes including resveratrol, flavonoids (such as flavonols, anthocyanins, and flavan-3-ols like catechins), melatonin, vitamins, acids, tannins, and other antioxidants (Figure 1).

These compounds have medical effects on diseases caused by oxidative stress. Free radicals play a pivotal role in various physiological processes and are involved in a wide range of diseases [5]. They are reactive chemical species with a single unpaired electron, making them chemically unstable and contributing to oxidative stress. In most cases, they are harmful to humans [6]. Oxidative stress is a condition in which the production of free radicals exceeds the body’s ability to neutralize them with antioxidants, resulting in cellular damage. They can trigger various diseases, including cancer, brain and eye diseases, and many other diseases [7,8,9,10]. Phenolic compounds can neutralize free radicals and may play an important role in preventing diseases caused by oxidative stress [11]. Grapes have many phenolic compounds, such as resveratrol, flavonoids, and tannins. Different grape varieties have different antioxidant activities [12,13].

The rich compounds in grapes provide numerous medical benefits beyond their traditional uses. In addition, natural biomaterials offer several advantages, such as non-toxicity, biocompatibility, and biodegradability. Due to the high demand for medical applications, the use of natural biomaterials has steadily increased [14]. Biomaterials, such as hydrogels extracted from grapes or their byproduct waste, have shown promising medical applications. This study reviews the medical effects of grape extracts and the biomedical applications of grape-derived biomaterials, aiming to inspire further potential uses of grapes in medical research and practice.

## 2. Varieties

The cultivation of domesticated grapes dates back 6000 to 8000 years in the Near East [15]. Grapes are cultivated for a variety of purposes and are commonly used as table grapes, wine grapes, and raisin grapes [16]. There are many grape species such as Vitis vinifera, Vitis labrusca, Muscadinia rotundifolia, Vitis riparia, Vitis aestivalis, Vitis amurensis, Vitis berlandieri, Vitis rupestris, etc. [17]. Among these, Vitis vinifera is the most common grape species used for both the production of wine and table grapes around the world, which originated in western Asia [18,19]. Other species are grown in specific regions and occupy smaller cultivation areas.

Vitis vinifera includes cultivars such as Thompson seedless, chardonnay, cotton candy grapes, and others [20,21]. Cotton candy grapes are a hybrid variety that is considered neutral in aroma [20]. Vitis labrusca include Catawba, Isabella, Concord, Delaware, Hartford prolific, and Diana and Niagara [22]. Vitis rotundifolia, commonly known as muscadine, is a grapevine species native to the southeastern and south-central United States [23]. In these areas, muscadines are highly valued for their distinctive flavor and aroma and are cultivated for juice, wine production, and fresh consumption [24]. Figure 2 displays various grape varieties. The varieties of grapes used for several purposes, including fresh consumption, as well as wine, raisin, juice, jam, and jelly production, are summarized in Table 1.

Winemaking is arguably the most significant industry associated with grapes. Yeast, one of the first domesticated microorganisms, naturally occurs on grape skins [15]. Yeast is responsible for the fermentation of grapes into wine, which led to the discovery of alcoholic beverages. The yeast ecology of grapes in China has been studied, and 17 different yeast species have been detected on the grapes [25]. Winemaking is an ancient yet continuously developing technique [26]. Grapes typically harbor a natural population of wild microorganisms. Different technologies can effectively eliminate or significantly reduce this wild microbiota, enabling more hygienic winemaking processes [27]. The management of grape-derived wine aroma also plays a crucial role in maintaining both the quality and shelf life of wine [28].

Raisins are another important byproduct of grapes. The consumption of raisins offers numerous health benefits, supporting overall wellness and helping to prevent various chronic conditions such as cardiovascular disease, type 2 diabetes mellitus, gastrointestinal disorders, and dental caries [29]. Along with wine production, the preparation of raisins provides an efficient way to preserve grapes during periods of overproduction.

Grapes are now cultivated worldwide. The top grape-producing countries include China, France, the United States, South Africa, Italy, Chile, Iran, Turkey, Spain, and Argentina [15]. The management process is crucial. Grapes are harvested, sorted to remove damaged or low-quality ones, and then distributed to markets, grocery stores, and wineries or exported to other countries. For efficient distribution, it is important to develop cool storage near vineyards and cooling systems throughout the supply chain to maintain optimal post-harvest temperatures for grapes [15]. Some methods have been proposed to evaluate the package, management, and supply chain of grapes, focusing on risk evaluation and management in the fresh grape supply chain to enhance its sustainability [30,31].

## 3. Bioactive Compounds

**Figure 3:**
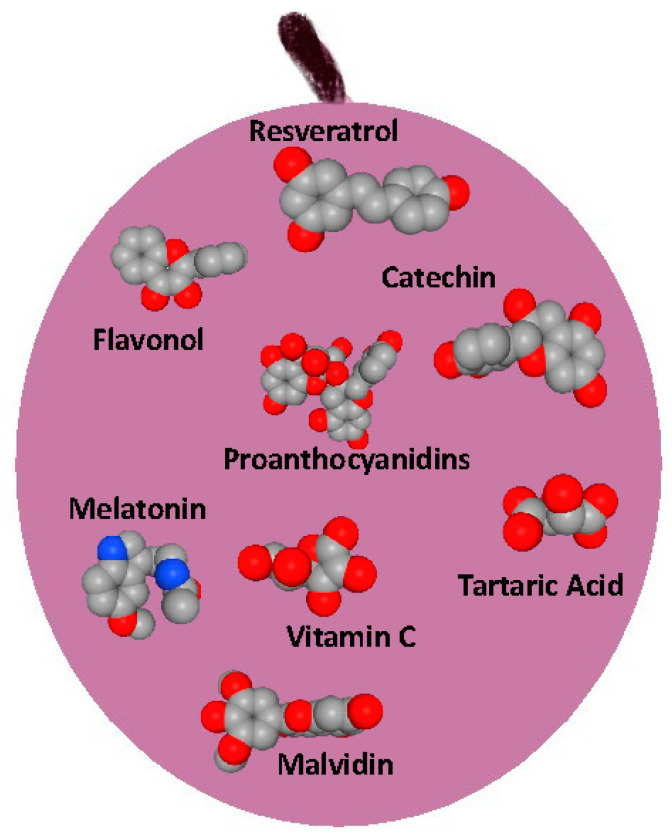
The chemical structures of several bioactive compounds in grapes, including resveratrol, flavonols, catechin, melatonin, malvidin, vitamin C, anthocyanins, tartaric acid, and proanthocyanidins.

As mentioned, grapes contain various bioactive compounds, including polyphenols such as resveratrol, flavonols, catechins, anthocyanins, and tannins, as well as melatonin, vitamins, acids, and other antioxidants, all of which contribute to health benefits (Figure 3). Grape waste, including peels and canes, is also rich in these compounds [32].

Phenolic compounds, including polyphenols, are natural phytochemicals and polyphenols are a class of semi-water-soluble substances [33]. In plants, polyphenols are special compounds that help protect plants by fighting off pests and diseases (biotic stress) and coping with environmental challenges (abiotic stress) [34]. These compounds are commonly found in foods such as fruits, vegetables, nuts, seeds, flowers, and tree barks, as well as in popular beverages like wine, beer, and tea, making them an essential part of the human diet [35,36]. Grapes are among the fruits rich in a variety of polyphenols. These polyphenols are primarily synthesized in the skin and seed tissues of grape berries and are extracted during the winemaking process [37].

Grape seeds and skins are rich in phytochemicals like gallic acid, catechin, and epicatechin, making them ideal raw materials for producing antioxidant dietary supplements [38]. Resveratrol, a key stilbene phytoalexin, is naturally synthesized or induced in plants as part of their defense mechanism. Grapes and their derivatives, such as juice and wine, are their primary natural sources of resveratrol [39]. Flavonoids are the largest group of grape polyphenols, which are found in grape skin or seeds [40]. Anthocyanins are natural colorants responsible for the color of red grapes and wines [41]. These polyphenols in grapes have antioxidant, cardioprotective, anticancer, anti-inflammatory, anti-aging, or antimicrobial properties [42]. In addition, tannins are a type of polyphenol commonly found in fruits, leaves, and trees. Grapes also contain tannins [43]. Tannins are used in the tanning process of the leather industry [44].

Melatonin is a hormone commonly used to address sleep problems, such as insomnia or disrupted sleep patterns [45]. Iriti, Rossoni, and Faoro first reported the presence of melatonin in grape extract from some Vitis vinifera cultivars [46,47]. Melatonin, first identified as a hormone of the pineal gland, is also produced by bacteria, protozoa, plants, fungi, invertebrates, and various extra-pineal sites in vertebrates, such as the gut, skin, Harderian gland, and leukocytes [48]. It is an ancient antioxidant that originated in bacteria and has been preserved throughout evolution without any changes to its chemical structure. The melatonin found in humans today is identical to that in cyanobacteria billions of years ago. In mammals, melatonin quickly disappears from the bloodstream as it is taken up by cells, particularly under oxidative stress. Its concentration in mitochondria is much higher than in blood. Mitochondria not only take up melatonin but may also produce it, as they evolved from melatonin-forming bacteria via the endosymbiotic theory. This localization supports melatonin’s role as a potent mitochondrial antioxidant, protecting against free radicals [49].

Grapes, like other fruits, are rich in vitamins. Five vitamins—A, B1, B2, B6, and C—were measured in Gök Üzüm raisins. Vitamin B2 was the most abundant, followed by vitamin B6, while vitamin C was the least abundant [50]. The waste from winemaking can be used to extract vitamins. Vitamin E can be extracted from grape seed oil derived from wine industry residues [51]. In recent years, the use of organic acids as alternatives to antibiotic growth promoters has increased due to concerns about antibiotic resistance and its potential impact on human health [52]. Grapes contain natural acids such as tartaric acid, malic acid, and citric acid. Tartaric and malic acids are the primary organic acids in grape juice, while succinic and citric acids are present in smaller amounts [53].

## 4. Medical Effects on Diseases

**Figure 4:**
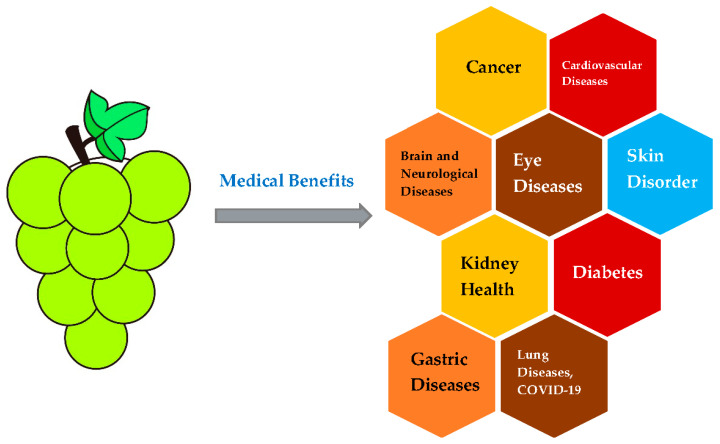
Medical effects of grapes on various diseases.

This paper reviews the medical effects of the compounds in grapes on various diseases (Figure 4).

### 4.1. Cancer

Cancer is a complex, multistep disease influenced by various factors, including environmental, chemical, physical, metabolic, and genetic elements. The beneficial role of polyphenol-rich foods in lowering cancer risk or mortality is well supported and undeniable [54].

Catechin hydrate has demonstrated anticancer potential, with the mechanism of action of many anticancer drugs relying on their ability to induce apoptosis. It exhibits anti-breast cancer effects by inhibiting the proliferation of MCF7 cells and triggering apoptosis [55]. Resveratrol has garnered significant scientific and public attention for its widely recognized potential to prevent cancer, slow aging, and mitigate associated metabolic diseases [39]. Grape seed extract (GSE) was effectively encapsulated in chitosan microparticles, which demonstrated significant anticancer activity [56].

Flavonoids are now regarded as essential components in various nutraceutical, pharmaceutical, medicinal, and cosmetic applications, owing to their antioxidant, anti-inflammatory, anti-mutagenic, and anti-carcinogenic properties, as well as their ability to regulate key cellular enzyme functions [57]. Natural polyphenols derived from grapes and wine have been studied in various breast cancer models, both in vivo and in vitro [58]. These compounds exhibit significant anticancer effects, including the modulation of estrogen signaling, promotion of cancer cell differentiation, inhibition of cell growth, induction of apoptosis, and suppression of metastatic behavior.

Preclinical or clinical trials have shown grape components to be promising therapeutic agents for cancer. Preclinical research on muscadine grape extract, derived from muscadine grape skins and seeds, has demonstrated anti-tumor effects and a reduction in systemic inflammation [59]. MPX, a powdered form of muscadine grape skin, contains ellagic acid, quercetin, and resveratrol and has shown preclinical activity against prostate cancer cells in vitro. Its safety and tolerability have been assessed in prostate cancer patients [60]. The red grape polyphenol resveratrol has shown potential as a therapeutic molecule for cancer in preclinical or clinical trials [61].

### 4.2. Cardiovascular Disease

Epidemiological studies indicate that consuming wine, grape products, and other polyphenol-rich foods is linked to a reduced risk of cardiovascular disease [62]. Polyphenol intake supports vascular health, which can significantly lower the risk of hypertension and cardiovascular disease [63].

Anthocyanins possess antioxidant, antimicrobial, and anticarcinogenic properties and provide protective benefits to the human cardiovascular system [41]. A GSE standardized for polyphenols from *Vitis vinifera* L. demonstrated benefits in enhancing endothelial function and regulating blood pressure [64].

The term French Paradox was introduced in 1992 to describe the relatively low rates of cardiovascular disease among the French population, despite their high dietary intake of saturated fats, possibly linked to red wine consumption. Consuming alcohol at the typical intake level in France (20–30 g per day) may lower the risk of coronary heart disease by at least 40% [65]. Components of red wine, particularly alcohol, resveratrol, and other polyphenolic compounds, may reduce oxidative stress and promote cholesterol efflux from vessel walls. Light to moderate red wine consumption generates a range of potentially beneficial effects that influence every stage of the atherosclerotic process, from atherogenesis to vessel occlusion [66]. In contrast, chronic heavy alcohol consumption and binge drinking are linked to a higher risk of cardiovascular events.

### 4.3. Brain and Neurological Disease

Brain and neurological diseases cover a broad spectrum of conditions, from lighter issues like sleep disorders to more severe disorders that lead to cognitive, motor, or sensory impairments. Regular consumption of polyphenol-rich foods may support brain health and potentially reduce the risk or progression of neurological disorders, including Alzheimer’s disease, Parkinson’s disease, cognitive decline, and even mild conditions like sleep disturbances [67]. The aggregation of amyloid-β peptide (Aβ) is associated with Alzheimer’s disease. Grape skin and seed extracts enhance the inhibition of Aβ aggregation. Solid lipid nanoparticles functionalized with an antibody offer a potential carrier for delivering the extracts to the brain [68].

Posttraumatic stress disorder (PTSD), a mental health condition triggered by extremely stressful or traumatic events, is twice as common in women as in men [69]. Grape powder has been demonstrated to prevent cognitive, behavioral, and biochemical impairments in a rat model of PTSD [70].

Sleep is a vital biological process, playing a crucial role in both physiology and behavior for maintaining quality of life. Consuming milk and sour cherries such as grapes, which are natural sources of melatonin, may enhance sleep quality in humans [71].

Melatonin is the primary hormone regulating the sleep–wake cycle and is often used to manage sleep issues, such as insomnia or disrupted sleep patterns [72,73]. Recently, melatonin has attracted significant attention primarily due to its potent lipophilic antioxidant properties and its ability to scavenge free radicals effectively [74]. Melatonin was initially proposed to function as a detoxifier of free radicals produced during photosynthesis and metabolism. As organisms evolved and diversified, melatonin developed into a pleiotropic molecule, not only counteracting oxidative stress but also regulating biological rhythms, suppressing inflammation, and more [75].

Resveratrol has been demonstrated to have good effects on sleep. Sleep deprivation impairs hippocampal synaptic plasticity and memory storage. A bioactive dietary polyphenol preparation (BDPP), containing GSE, concord grape juice, and resveratrol, was tested for its ability to reduce cognitive deficits caused by sleep deprivation [76]. BDPP compounds showed potential as therapeutic agents to counteract these effects.

### 4.4. Eye Diseases

In ancient Egypt, grapevine sap was used to create an ointment for treating skin and eye conditions [62]. Age-related eye diseases such as macular degeneration and glaucoma are degenerative retinal conditions and major contributors to irreversible blindness in developed countries [8,77]. Retinal degenerative conditions encompass a diverse group of diseases, with genetic mutations and susceptibility factors playing key roles in determining their onset and progression [78]. Grape juice has been widely used around the world for its powerful medicinal benefits, including supporting eye health [79]. A study investigated whether freeze-dried grape powder could enhance photoreceptor survival in a mouse model of retinal degeneration [80]. The results showed that a grape-supplemented diet preserved retinal structure and function in a mouse model of retinal degeneration caused by oxidative stress, highlighting the beneficial effects of grapes.

Diabetic retinopathy is a major cause of vision loss in working-age adults in industrialized areas. Since it shows no symptoms in the early stages, regular eye screenings for diabetes patients are essential for early detection and treatment [81]. Oxidative stress plays a role in the development of diabetic retinopathy [82]. Current treatments for diabetic retinopathy do not target oxidative stress directly. The effectiveness of a grape pomace extract, an oxidative stress inhibitor, for managing diabetic retinopathy has been examined [83]. The results showed that this supplementation effectively reduced retinal swelling and oxidative stress, leading to better visual outcomes in patients with diabetic retinopathy.

### 4.5. Skin Disorder and Wound Healing

Skin injuries, such as wounds, UV exposure, and excessive inflammation, can contribute to chronic dermatological disorders, which often require prolonged treatment. The use of berries for skin protection is rooted in long-standing traditional practices. Promising in vitro and in vivo evidence suggests that *Vitis vinifera* L. supports wound healing and offers photoprotection [84]. Angiogenesis plays an important role for wound healing, with the vascular endothelial growth factor (VEGF) being the most significant and effective growth factor promoting this process. Grape seed proanthocyanidin extract can enhance wound healing by increasing VEGF expression, promoting angiogenesis, and improving tissue structure [85].

GSE and its primary active polyphenol, resveratrol, demonstrate significant antioxidant properties, along with protective and therapeutic effects against various skin conditions [86]. The antioxidants in grapes protect the skin from harmful free radicals and help reduce the appearance of dark spots and wrinkles.

GSE from Marselan and Obeidi varieties had high phenolic content and catechins. Poly(ethylene glycol)-b-poly(lactide-co-glycolide) nanocarriers loaded with GSE significantly inhibited B16-F10 melanoma cell proliferation [87].

Atopic dermatitis (AD) is a chronic inflammatory skin condition that poses substantial health and economic challenges. Dietary grape powder (GP) alleviated AD-like symptoms in NC/NgaTndCrlj mice with 2,4-dinitrofluorobenzene (DNFB)-induced AD [88]. GP supplementation effectively inhibited DNFB-induced AD in these mice during both prevention and intervention trials.

### 4.6. Kidney Health

Chronic kidney disease (CKD) is a progressive, incurable condition with high morbidity and mortality rates. It commonly occurs in the general adult population, particularly in individuals with diabetes or hypertension [89].

Patients with CKD are at high risk for cardiovascular disease, which can potentially progress to end-stage renal disease [90]. Oxidative stress plays a role in CKD pathophysiology, and grape-based antioxidants may prevent the development of CKD and cardiovascular disease [90].

An interventional study was conducted on CKD patients, involving supplementation with a GSE capsules group and a placebo group [91]. Taking 2 g of GSE daily for six months improved certain kidney function parameters in CKD patients. Post-treatment with GSE boosted antioxidant defenses and increased Bcl-2 expression in mouse kidneys, suggesting its potential for developing treatments or prevention strategies for hyperthyroidism-related kidney injury [92].

Regular long-term consumption of grapes supports kidney health and helps protect against fibrosis and related conditions in a C57BL/6J mice study [93]. The daily consumption of grape powder prevents the progression of kidney disease in obese type 2 diabetic ZSF1 rats [66].

### 4.7. Diabetes

Diabetes mellitus is an endocrine disorder caused either by insufficient insulin production by the pancreas or by the body’s inability to respond effectively to insulin [94]. The loss of functional β-cell mass is the primary mechanism driving the development of the two major forms of diabetes mellitus: type 1 diabetes mellitus and type 2 diabetes mellitus [95].

Several compounds in grapes such as the stilbene resveratrol, the flavanol quercetin, catechins, and anthocyanins have demonstrated the potential to reduce hyperglycemia, enhance β-cell function, and prevent β-cell loss [96]. A systematic review and meta-analysis of randomized clinical trials showed that grapes/grape products might have beneficial effects on the homeostatic model assessment of insulin resistance for adults [97]. In addition, tannins in the grapes may help reduce the risk of diabetes by promoting glucose uptake and lowering blood sugar levels [42].

### 4.8. Gastric Diseases

Gastric diseases are disorders affecting the stomach, including gastritis, ulcers, reflux, and cancers, often causing pain, indigestion, or nausea. A ten-day pretreatment with proanthocyanidin-rich GSE was more effective than vitamins E and C in reducing gastric lesions [98]. GSEs demonstrated a protective effect against acute gastric lesions in rats [99]. This protective effect on gastric mucosal injury is attributed to procyanidin oligomers. Acute and chronic stress can trigger gastrointestinal oxidative stress and mucosal damage by increasing the production of reactive oxygen species (ROS). Grape seed proanthocyanidin extract offers significant protection against this oxidative stress and mucosal injury by scavenging these ROS [100].

*Helicobacter pylori* (*H. pylori*) is a pathogenic bacterium recognized as a potential risk factor for gastritis, gastric ulcers, and gastric cancer. The effectiveness of a procyanidin-enriched GSE in combating H. pylori infection has been demonstrated [101]. Grape seed powder enhanced the intestinal transit rate in both normal mice and mice with acetic acid-induced gastrointestinal motility disorders [102].

### 4.9. Lung Diseases and COVID-19

Common lung diseases include pneumonia, asthma, sleep apnea syndrome, interstitial pneumonia, lung cancer, and chronic obstructive pulmonary disease [103]. The consumption of grape phytochemicals and vitamin D helps slow the progression of lung diseases. Both phytochemicals and vitamin D inhibit the production of the proinflammatory cytokine TNF-α, which contributes to inflammation and lung disease [103].

Coronavirus disease 2019 (COVID-19), caused by severe acute respiratory syndrome coronavirus 2 (SARS-CoV-2), has significantly impacted daily life, the economy, and livelihoods for several years [104,105]. Grapevine extract has been demonstrated to have antiviral activity which is primarily attributed to its ability to disrupt the cell membrane and inhibit the early stages of the viral life cycle, including attachment and fusion to the host cell [106]. Leaf extract effectively inhibited the replication of both Herpes simplex virus type 1 and SARS-CoV-2 in the early stages of infection by directly blocking viral surface proteins, even at a low concentration of 10 μg/mL [106]. Resveratrol, which is abundant in red grapes, has demonstrated strong in vitro inhibition of SARS-CoV-2, while resveratrol–zinc nanoparticles offer a significant pharmacokinetic advantage for COVID-19 treatment [107]. A randomized, double-blind, placebo-controlled clinical trial demonstrated the beneficial effects of almond porridge, grape extract, and pea syrup in reducing fatigue severity and improving clinical symptoms in COVID-19 patients experiencing high levels of fatigue [108]. The water extract of grape seeds was rich in oligomeric proanthocyanidins, which, along with punicalagin, demonstrated inhibitory activity against Omicron variants of SARS-CoV-2 infection [109].

## 5. Medical Polymer

**Figure 5:**
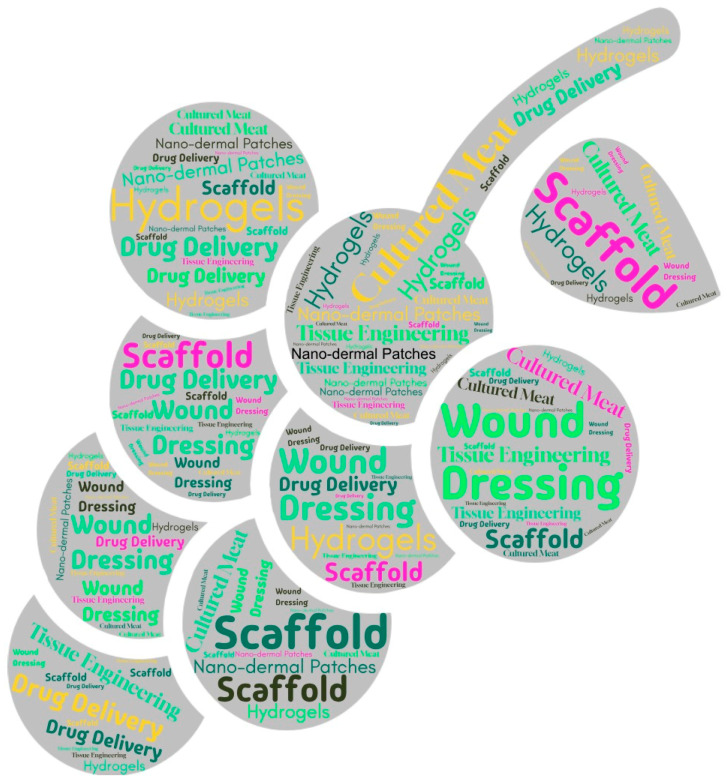
The medical polymer applications of grapes.

This paper reviews the medical polymer applications of grapes (Figure 5).

### 5.1. Drug Delivery

Drug delivery is a technology used to administer therapeutic substances to achieve optimal effects in the body. Drug delivery systems (DDSs) refer to methods that transport drugs to specific tissues, organs, cells, or subcellular targets, using various carriers to facilitate drug release and absorption [110]. Cell and gene therapies are advanced therapeutic delivery methods, while nanoparticles play a crucial role in enhancing and refining DDSs [111].

GSEs have beneficial applications in DDSs. The development of nanofibrous membranes with tunable wettability, degradation, and biocompatibility is highly keen for biomedical applications, including drug delivery and wound dressing. Locilento et al. (2019) created nanofibrous membranes with antioxidant properties using polylactic acid and polyethylene oxide (PEO) as the matrix, enhanced with GSE [112]. The results indicated that the GSE-loaded membranes are a promising topical DDS.

Components derived from grapes, such as polysaccharides and polyphenols, have been used in the development of hydrogels. Hydrogels are three-dimensional, cross-linked structures made from natural or synthetic polymers. Their unique properties, such as a high swelling capacity and permeability to hydrophilic substances, have garnered significant interest. Hydrogel-based carrier systems play a vital role in drug delivery applications [113].

Vitis vinifera fluid extract from leaves was combined with silica to form nanocomposites, which are then used in a DDS, particularly for wound healing [114]. Grape skin, stalk, and seeds left over from grape processing can be utilized to produce gold nanoparticles (AuNPs) from chloroauric acid $\left({HAuCl}_{4}\right)$ [115]. The average particle size of green-synthesized gold nanoparticles derived from grape waste ranged between 20 and 25 nm. The water-dispersible nature of gold nanoparticles derived from grape byproducts, combined with their inherent health benefits, has the potential to revolutionize nanotechnology’s impact on drug and nutraceutical delivery.

### 5.2. Wound Dressing

Wound management poses a significant global challenge, placing a substantial financial burden on governments worldwide. Wound dressings that promote healing have been the focus of research for an extended period [116]. Traditional wound dressings primarily serve as passive barriers, offering environmental protection without actively promoting healing and relying on external factors to address infections.

Hydrogels, with their outstanding biochemical and mechanical properties, offer significant advantages in the field of wound dressings [117]. White grape pomace extracts were incorporated into a Chitosan/Alginate hydrogel, which demonstrated high swelling ability and antibacterial activity against *S. aureus* [118]. Using PLA and PEO as the matrix, with added GSE, the GSE-loaded PLA/PEO membranes exhibited better cell attachment and proliferation compared to PLA/GSE membranes, due to superior hydrophilicity [112]. These GSE-loaded membranes not only show great promise as a topical DDS, but also have significant potential for wound dressing applications.

Nano-dermal patches have gained attention for offering higher surface energy, deeper penetration, and increased entrapment sites compared to traditional ones. A nanomembrane incorporating Vitis vinifera extract and polyvinyl alcohol (PVA) through the electrospinning method has been developed [119]. The nanomembranes exhibit up to 79% antioxidant activity and 99.8% antibacterial effectiveness against Staphylococcus aureus (*S. aureus*) [120]. Lin et al. (2016) developed a GSE-loaded silk fibroin/PEO composite nanofiber using green electrospinning [121]. They highlighted the potential of these GSE-loaded nanofibrous mats for applications in skin care, tissue regeneration, and wound healing.

### 5.3. Tissue Engineering

Tissue engineering is a field within biomedical engineering that combines cells, engineering techniques, materials science, and appropriate biochemical and physicochemical factors to restore, maintain, enhance, or replace various types of biological tissues. Regenerative medicine strategies focus on restoring damaged tissue structures using cell transplantation with scaffolds and biomolecules. Biomaterial scaffolds, such as natural and synthetic polymers, bioresorbable materials, and decellularized tissues, offer enhanced structural, mechanical, and biological properties [122].

Plant-derived porous hydrogels offer more benefits than traditional hydrogels when used as chromatographic materials, controlled release carriers for drugs and proteins, matrices for immobilizing or separating molecules and cells, or as scaffolds in tissue engineering [123]. Polyphenols are a group of natural compounds known for their numerous health benefits, including bioadhesive, antioxidant, and antibacterial properties. The development of polyphenol-based biomaterials represents a major advancement in promoting various health-related applications such as tissue engineering and bone regeneration [124]. GSEs are rich in flavonoids, including oligomeric proanthocyanidins (OPCs). Hydrogel scaffolds containing OPC serve as natural photothermal agents for melanoma therapy and bioactive materials for wound healing [120]. The controlled high temperatures generated by OPC-containing hydrogels under near-infrared laser irradiation effectively eliminate melanoma cells and inhibit tumor growth.

Tissue-engineered skin offers a promising alternative to traditional wound dressings. Additionally, fibrous scaffolds created using textile technologies are highly appealing for tissue engineering. The development of electrospun polyurethane textile wound dressings infused with grape extract has been investigated [125]. The incorporation of grape extract into the polyurethane matrix improved the physicochemical properties and biocompatibility, positioning it as a promising candidate for skin tissue engineering and regeneration.

Dental caries is the most common chronic disease globally. Resin composites are commonly used to restore tissue loss caused by caries, but their lifespan is limited to a few years. Recently, plant-derived extracts rich in proanthocyanidins (PAs), such as GSEs, have been shown to enhance the mechanical properties and biostability of the dentin matrix, as well as improve dentin–resin bond strength [126]. Additionally, the potential impact of proanthocyanidins from GSEs on caries-affected dentin was examined [127]. The organic matrix of dentin primarily consists of fibrillar type I collagen and non-collagenous proteins. Proanthocyanidins are recognized as natural cross-linking agents for collagen in the dentin matrix, as they enhance hydrogen bond formation in type I collagen.

Essential oils are significant in alleviating pain and inflammation associated with bone fractures. An electrospun scaffold was developed using polyurethane (PU), grape seed oil, honey, and propolis for bone tissue engineering applications [128]. The fabricated PU/grape seed oil and PU/grape seed oil/honey/propolis scaffolds exhibited improved thermal stability and reduced surface roughness compared to the control. Additionally, the scaffolds demonstrated prolonged blood clotting time, indicating enhanced blood compatibility relative to the PU control.

Cell-cultured meat, produced through the in vitro cultivation of animal-derived cells, has gained significant attention as a potential protein source for the future. Porous hydrogels play a vital role as artificial extracellular matrices (ECMs), supporting cell growth for the production of cultured meat. The impact of GSE (proanthocyanidins, PC) on the physical, chemical, and biological properties of an edible gelatin-based hydrogel, including bovine satellite muscle cell growth and adhesion, has been studied [129]. It suggested that hydrogels developed from edible materials could support muscle cell growth for cultured meat production.

## 6. Discussion

### 6.1. Dietary Restrictions

A global increase in diet-related chronic diseases has prompted efforts to formally incorporate nutritious food into patient care and treatment. A poor diet is now responsible for one in every five deaths globally, surpassing all other risk factors, including tobacco use [130]. Grapes and their byproducts have been demonstrated to have many medical effects which might be recommended to prevent, manage, and treat certain illnesses. While grapes have many medicinal benefits, the direct consumption of some grape varieties may be a concern for some people.

Potassium is the most abundant positively charged ion in the grape berry [131]. Potassium is known to benefit kidney health. In the early stages of CKD, high potassium intake typically does not cause hyperkalemia, unless certain treatments are used. High potassium diets can be beneficial for CKD patients, supporting cardiovascular and renal health. However, in advanced CKD and end-stage renal diseases, excessive potassium intake can lead to hyperkalemia, requiring careful management of dietary potassium [132]. Therefore, individuals with CKD should exercise caution with grape consumption.

Grapes contain organic acids such as malic, tartaric, and acetic acids. The acidity of grapes at harvest is influenced by the levels of organic acids and potassium. Malic and tartaric acids account for up to 90% of the total organic acids present in grapes [133]. Acetic acid is present in grapes but in much smaller quantities. Acetic acid might worsen mouth ulcers [134]. Therefore, for people with mouth ulcers, the acids in grapes might irritate the open sores when consuming grapes or wine directly.

Gastroesophageal reflux disease (GERD) is characterized by recurrent and troublesome heartburn and regurgitation, or GERD-specific complications, affecting approximately 20% of adults in high-income countries [135,136]. Factors triggering GERD symptoms include fatty, fried, sour, spicy food/products, juice, coffee/tea, carbonated beverages, and alcohol [137]. Acidic foods such as sour fruit grapes might cause GERD symptoms.

Grapes contain higher levels of oxalates compared to some other fruits. A study indicated that among various fruit juices, red grape juice had the highest concentrations of both soluble and total oxalates [138]. Kidney stone disease involves the formation of crystalline deposits, typically within the kidneys. It is a growing urological concern, affecting approximately 12% of the global population [139]. The most prevalent type of kidney stone is calcium oxalate, which commonly forms on Randall’s plaques located on the renal papillary surfaces. Dietary oxalate intake significantly influences the risk of kidney stone formation [140]. For individuals prone to kidney stones, excessive grape consumption may be a concern.

### 6.2. Limitations and Future Research Perspectives

While grape-derived compounds have medical applications in drug delivery, wound dressing, and tissue engineering, there are still obstacles and constraints in these applications.

Bioavailability refers to the degree to which a substance or drug is fully accessible to its intended biological target(s) [141]. Polyphenols generally have low bioavailability, influenced by various factors such as interactions with the food matrix, metabolic processes in the liver, as well as the role of the intestine and microbiota [142].

**Figure 6:**
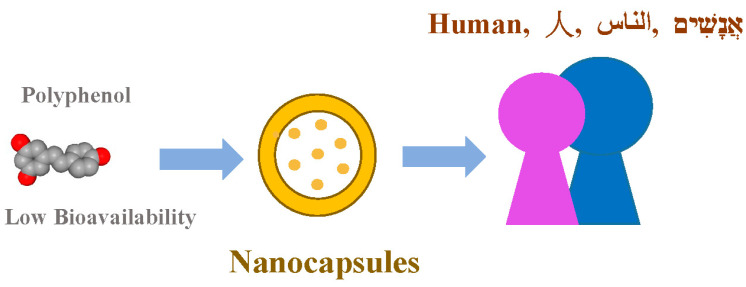
Nanoencapsulation techniques can be used to improve the stability of polyphenols.

To enhance the bioavailability and stability of grape-derived compounds, nanoencapsulation techniques can significantly improve the stability of these bioactive compounds, particularly resveratrol [143] (Figure 6). Targeted drug delivery systems can be developed using nanoparticles or hydrogels [144,145]. Hydrogels serve as soft, biocompatible carriers for delivering plant-derived (poly)phenols—compounds with low overall toxicity and a remarkable yet partially unexplored diversity of biological properties [145].

Compared to synthetic polymers, natural polymers are biodegradable, eco-friendly, and have antimicrobial capacity [146,147,148]. While synthetic polymers may offer durability, tunable properties, and endless forms, which can help restore damaged or diseased tissue structure and function [149], they may lack some beneficial properties found in natural polymers. Conventional synthetic polymers are often non-biodegradable, and they often lack biocompatibility, which may pose health risks or cause adverse effects. Biodegradable synthetic polymers have been developed and are widely used in medical applications [150,151].

To balance the advantages and limitations of natural polymers, the combination of synthetic and grape-based polymers in medical applications is an emerging area of research and development. These hybrid materials integrating the durability and processability of synthetic polymers with the biocompatibility and biodegradability of grape-based polymers may offer promising potential for biomedical use.

The C-reactive protein (CRP) is an acute-phase protein produced by liver cells in response to inflammation [152]. A meta-analysis showed that higher consumption of grape products containing polyphenols was linked to reduced concentrations of CRP [153]. However, the safety of high doses of polyphenols for humans is still unknown [154]. Finding the right dose for therapeutic effects without toxicity remains challenging. Since effects may differ between individuals due to genetics, metabolism, or existing health conditions, personalized grape-derived medicine can be studied.

## 7. Conclusions

Grapes are a fruit originating from the ancient age. The first use of grapes recorded in the Bible is winemaking. The riches of bioactive compounds in grapes make them a valuable fruit. This paper reviews the medical effects of these compounds on cancer, cardiovascular disease, brain and neurological diseases, eye diseases, skin disorders, kidney health, diabetes, gastric diseases, lung diseases, and COVID-19, as well as the medical applications of grapes, including drug delivery, wound dressing, and tissue engineering. These benefits demonstrate that the value of grapes extends beyond their traditional role in wine and raisin production.

## Figures and Tables

**Table 1:** Grape varieties for four different uses.

Uses	Variety
Fresh Consumption	Thompson Seedless, Cotton Candy, Isabella, Concord, Niagara, Muscadine
Wine Production	Thompson Seedless, Chardonnay, Catawba, Isabella, Concord, Niagara, Muscadine
Raisin Production	Thompson Seedless
Juice, Jam, Jelly	Catawba, Isabella, Concord, Niagara, Muscadine
